# Is colorectal mucosa a reasonable graft alternative to buccal grafts for urethroplasty?: A comparison of graft histology and stretch

**DOI:** 10.1590/S1677-5538.IBJU.2022.0278

**Published:** 2022-10-25

**Authors:** Jane T. Kurtzman, Christopher Sayegh, Shawn Mendonca, Mahveesh Chowdhury, Preston Kerr, Carlos Pagan, Marco B. Zoccali, Steven B. Brandes

**Affiliations:** 1 Columbia University Irving Medical Center Department of Urology New York NY USA Department of Urology, Columbia University Irving Medical Center, New York, NY, USA; 2 Columbia University Irving Medical Center Department of Pathology and Cell Biology New York NY USA Department of Pathology and Cell Biology, Columbia University Irving Medical Center, New York, NY, USA; 3 Columbia University Irving Medical Center Division of Colorectal Surgery Department of Surgery New York NY USA Division of Colorectal Surgery, Department of Surgery, Columbia University Irving Medical Center, New York, NY, USA

**Keywords:** Urethral Stricture, Mouth Mucosa, Reconstructive Surgical Procedures

## Abstract

**Objective:**

To compare the histological properties and stretch of colorectal mucosal grafts (CMG) and buccal mucosal grafts (BMG) and to evaluate the impact of age, medical comorbidity and tobacco use on these metrics.

**Materials and Methods:**

Samples of BMGs from patients undergoing augmentation urethroplasty were sent for pathologic review. CMGs were collected from patients undergoing elective colectomy. CMGs were harvested fresh, at full thickness from normal rectum/sigmoid. Patients with inflammatory bowel disease, prior radiation, or chemotherapy were excluded.

**Results:**

Seventy two BMGs and 53 CMGs were reviewed. While BMGs and CMGs were both histologically composed of mucosal (epithelium + lamina propria) and submucosal layers, the mucosal layer in CMG had crypts. The outer epithelial layers differed significantly in mean thickness (BMG 573μm vs. CMG 430μm, p=0.0001). Mean lamina propria thickness and submucosal layer thickness also differed significantly (BMG 135μm vs. CMG 400μm, p<0.0001; BMG 1090μm vs. CMG 808μm, p = 0.007, respectively). Mean delta stretch, as to length and width, was greater for CMG (118% x 72%) compared to BMGs (22% x 8%), both p<0.001.

**Conclusion:**

CMGs and BMGs significantly differ histologically in layer composition, width and architecture, as well as graft stretch. Given its elastic properties, CMG may be useful in covering large surface areas, but its thin epithelium, thick lamina propria and additional muscularis mucosal layer could impact graft take and contracture.

## INTRODUCTION

The buccal mucosal graft (BMG) is the most widely used graft for urethral reconstruction (1). Unique qualities of BMG offer many advantages to the reconstructive urologist. The BMG is accustomed to a wet environment, it is hairless, easy to harvest, and the donor site is hidden with minimal procedure morbidity. It has a thick, elastin-rich epithelial layer, that makes it easy to handle, durable, resistant to infection and less likely to contract. In addition, its lamina propria is thin and highly vascular, which has been proposed to help facilitate efficient imbibition and inosculation resulting in excellent graft ‘take’ (2–7). BMGs also fully integrate into the corpus spongiosum, while retaining their original histologic cell types-unlike preputial and tunica vaginalis grafts (8).

Certain patients may have specific relative or absolute contraindications to BMG use, such as: prior BMG harvest, surgery or radiotherapy for oral cancer treatment, active tobacco use or oral mucosal diseases. Poor oral health has also been shown to impact BMG histologic characteristics (9). Moreover, for many pan-urethral strictures or long obliterative strictures there may be insufficient total oral graft material for reconstruction (10). Therefore, surgeons must always consider both clinical factors and stricture characteristics before proceeding with surgery (11).

As an alternative to buccal grafts, colorectal mucosal grafts (CMGs) can be utilized and have recently been shown, in several small series, to successfully treat complex long urethral strictures (12–15). Although CMG outcome analysis is limited by follow-up length, the success rates seem to be similar with both grafts (2, 12, 13, 15, 16). At first glance, CMGs seem to have many of the same advantages as BMGs – they are both wet mucosa, hairless and acquired from a hidden donor site. CMGs are relatively easy to harvest using a trans-anal endoscopic microscopy (TEM) resectoscope but however, this require unique technical skill. TEM surgery carries up to a 30% risk of complications including: bleeding, perforation, fecal incontinence, and rectal stenosis (17). However, this risk been reported to be as low as 0% for CMG harvest in urologic surgery (15). In comparison, harvesting BMG grafts carries risks of bleeding, scar bleeding, scar contracture, difficulty with mouth opening and decreased oral sensation (15).

While both grafts have demonstrated clinical applicability, it is unknown how their physical and histologic properties compare. These characteristics are important to analyze as they may favor the ‘take’ of one graft over the other in urethral reconstruction. Therefore, the purpose of this study was to compare the histological properties and stretch characteristics of CMGs and BMGs. We hypothesized that CMG and BMGs would differ in cell layer thicknesses and in graft stretch.

## MATERIALS AND METHODS

We conducted a prospective analysis of all patients who underwent BMG urethroplasty at our institution and consented to participate. CMGs were collected from all consenting patients who underwent elective colectomy or proctectomy. Colorectal patients with inflammatory bowel disease, prior radiation, or chemotherapy were excluded. All surgeries were performed between 2018 and October 2021. This study was approved by the institutional review board (AAAS3576).

The protocol and technique for BMG harvest has been previously described and was standardized across all patients (9). An ovoid graft measuring approximately 5 × 2 cm was measured and marked on the inner cheek. Graft size was standardized to limit variables that could confound graft stretch metrics. The buccal submucosa was infiltrated with 10 mL 1% lidocaine with epinephrine. The graft was sharply harvested from the inner cheek superficial to buccinator muscle. Grafts were defatted on the back table with intent to have a macroscopic whitish appearance, consistent with Group 2 dissections described by Cavalcanti et al. (18). This dissection optimizes the balance between subepithelial connective tissue preservation and adipose and muscle tissue removal (18).

Graft measurements were taken with the graft on a silicone block, both on and off stretch. Excess BMG not needed for urethral reconstruction was sent to pathology for analysis. The defect in the mouth after graft harvest was measured and recorded.

Colectomy/proctectomy was performed by a board-certified colorectal surgeon and immediately sent to pathology. All CMG specimens were obtained within six hours from resection and not contaminated by formalin. A full-thickness 5 × 2 cm was measured and marked by a member of the research team, along a segment of palpably and visibly normal colon or rectum. The graft was excised, and the mucosa was dissected off the underlying muscularis propria layer. Unlike BMGs, there was no grossly visible layer that needed to be defatted. CMG dimensions were obtained in a similar fashion to BMGs, with the graft placed on a silicone block, on and off stretch.

Histological review of all grafts was performed by a single staff pathologist. Tissue was sent in 10% buffered formalin, grossed, and embedded in paraffin block. Hematoxylin and eosin (H&E) staining was performed on 5-μm tissue sections on glass slides. A minimum of 10 high power fields were examined for each prepared slide. Measurements were taken from three 100x fields using an Olympus (Japan) BX41 microscope using a U-OCM10/100 eyepiece reticle 1 mm micrometer. Digital images were taken with an Olympus QColor3 camera using QCapture software (Tokyo, Japan).

Average epithelial, lamina propria, and submucosal thickness were measured and recorded. A modified version of the previously established Oral Mucosa Rating Scale was used to quantify the type and severity of pathological mucositis for all grafts. This scale was originally developed to assess for the severity, on a scale of 0 to 3, of seven types of clinical mucosal changes considered to be manifestations of clinical oral mucositis (19).

Graft stretch was assessed using the following formula: (Ds-Dd)/(Dd)*100%. Where Ds is equal to the dimension (length or width) of the graft stretched on the silicone block after macroscopic defatting (for BMGs) or muscularis propria removal (for CMGs) and Dd is equal to the corresponding dimension (length or width) of the graft defect. Graft stretching was performed manually by using pins to secure the grafts on a block. Force required to stretch the graft over a unit of length was not measured or calculated.

Chi-squared and Fisher's exact tests were used to assess for differences in the clinical characteristics between BMG and CMG patients. Two-tailed t-tests were used to compare means and differences in graft characteristics, including stretch and histologic metrics. Individual multivariable linear regression models were used to evaluate the association between three hypothesis-driven patient-level covariates (age, tobacco use and Charlson Comorbidity Index score (CCI)), and graft characteristics for each graft type (BMG versus CMG). Statistics were performed using Stata/IC v16.1 (StataCorp LLC, College Station, Texas).

## RESULTS

Seventy-two BMGs and 53 CMGs were harvested. Table-1 displays cohort characteristics. BMG patients were significantly younger than CMG patients (mean 47.8 years vs. 65.7 years, p <0.001). CMG patients were more likely to have a significant history of tobacco use (22% vs. 17%, p = 0.05) and were generally less healthy as evidenced by the CCI (mean CCI of 3.5 vs. 1.6, p < 0.001). 28% of the colon specimens were ascending colon, 2% transverse, 53% sigmoid and 17% rectum. 36% underwent colectomy for diverticulitis, 51% for cancer, and 13% for other causes.

**Table 1:** Cohort Characteristics.

			Colorectal grafts	Buccal graft	P-value
Cohort size, n			53	72	
Age, mean (SD); [median]			65.7 (14.8); [69]	47.8 (17.1); [44]	p < 0.001
Tobacco use history, n (%)			22 (42)	17 (24)	p = 0.05
	Current, n (%)		22 (42)	5 (7)	p < 0.001
	Former, n (%)		0 (0)	12 (17)	
	Never, n (%)		31 (58)	51 (71)	
Unknown, n (%)			0 (0)	4 (5)	
Prior Chemotherapy, n (%)			2 (4)	1 (1)	p = 0.39
Age-Adjusted CCI, mean (SD); [median]			3.5 (2.1); [4]	1.6 (2.3); [0]	p < 0.001
Age-Adjusted CCI ≤ 3, n (%)			26 (49)	59 (82)	p < 0.001
Hypertension, n (%)			29 (55)	28 (39)	p = 0.08
Diabetes, n (%)			12 (23)	7 (9)	p = 0.05
Colorectal Graft Location, n (%)				–	–
	Ascending/Transverse/Descending		16 (30)	–	–
		Sigmoid	28 (53)	–	–
		Rectum	9 (17)	–	–

**Figure 1:**
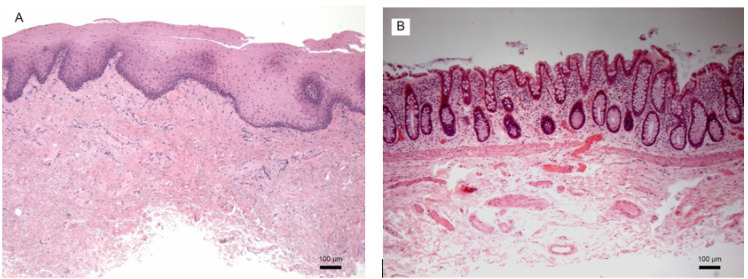
Images of buccal mucosal graft histology (A) and colonic mucosal graft histology (B).

Figure-1 displays a histologic sample of a typical BMG (Figure-1A) and CMG (Figure-1B) The BMG's epithelial layer was composed of a non-keratinizing stratified squamous epithelium. A dense blood supply of capillaries, venules and lymphatics were visualized within the lamina propria (LP). The deeper submucosa layer consisted of mainly connective tissue and fat with a less densely packed blood supply. The CMG's epithelial layer on the other hand was arranged in a cryptic architecture with a simple columnar epithelium supported by a LP. The LP of the CMG also included a dense supply of venules, capillaries, and lymphatics. However, it also contained many inflammatory cells and mucin-secreting goblet cells. Like BMG, the CMG submucosa was composed of connective tissues with additional vasculature.

Table-2 displays the histologic and stretch metrics of each graft type. BMGs and CMGs were found to have similar overall graft thickness (1798μm vs. 1667μm, p = 0.27). However, BMGs were found to have a significantly thicker epithelium (EP), 573μm vs. 430μm (p = 0.0001), a thinner lamina propria (LP), 135μm vs. 400μm, (p < 0.0001), and a thicker submucosal layer, 1090μm vs. 808μm (p=0.007). A muscularis mucosa layer with a mean thickness of 44μm was present only in CMGs. CMGs were found to have a significantly greater stretch ability by length (delta stretch 118% vs. 22%, p <0.0001) and by width (delta stretch 72% vs. 8%, p <0.0001).

**Table 2:** Graft Metrics.

		Colorectal grafts	Buccal grafts	P-value
**Histologic Layers, mean (SD); [median]**				
	Total graft thickness (microns)	1667 (583); [1550]	1797 (689); [1780]	p = 0.27
	Epithelial thickness (microns)	430 (110); [420]	573 (232); [535]	p = 0.0001
	Lamina propria thickness (microns)	400 (99); [390]	135 (80); [120]	p < 0.0001
	Muscularis mucosa thickness (microns)	44 (21); [40]	0 (0); [0]	p < 0.0001
	Submucosal thickness (microns)	808 (486); [655]	1090 (611); [1115]	p = 0.007
	Mucositis score	0 (0); [0]	0.8 (1.5); [1]	p = 0.0001
**Size and Stretch Characteristics, mean SD; [median]**				
	Length of excision site (cm)	5.7 (1.0); [5.5]	4.7 (1.0); [4.7]	p < 0.0001
	Width of excision site (cm)	2.3 (0.4); 2.1]	2.0 (0.4); [2]	p = 0.002
	Length of tissue sample (cm)	10.4 (3.5); [9.5]	4.8 (1.1); [4.75]	p < 0.0001
	Width of tissue sample (cm)	2.8 (0.8); [2.8]	1.7 (0.3); [1.7]	p < 0.0001
	Length of tissue sample on stretch (cm)	12.2 (3.4); [11.5]	5.7 (1.3); [5.55]	p < 0.0001
	Width of tissue sample on stretch (cm)	3.8 (1.0); [3.5]	2.2 (0.5); [2]	p < 0.0001
	Delta Stretch Lengthwise, (% change)	118% (57); [114%]	22% (20); [20%]	p < 0.0001
	Delta Stretch Widthwise, (% change)	72% (49); [59%]	8% (24); [0%]	p < 0.0001

Table-3 displays the results of individual multivariable linear regression models that evaluated the associations between the covariates of age, tobacco use, and CCI with graft characteristics for each graft type. Age, tobacco use, and CCI were not significantly associated with delta stretch (lengthwise or width wise) of both CMGs and BMGs (all p values > 0.05). However, increasing age was inversely correlated with epithelial thickness of BMGs (p = 0.003). This relationship was not evident with CMGs. Although tobacco use had no associated correlation with epithelial thickness of either graft type, we found a significant positive correlation between tobacco use (either former or current) and LP thickness (p = 0.035) in CMGs. We did not identify any associations between the three covariates and the submucosal thickness of either graft type.

**Table 3:** Results of Individual Multivariable Linear Regression Models to Evaluate Associations between Patient Level Covariates and Graft Characteristics.

		Colorectal Grafts		Buccal Grafts	
		Coeff	P-value	Coeff	P-value
**Stretch Lengthwise**					
	Age	-0.01	0.28	0.002	0.51
	Tobacco	-0.12	0.50	-0.04	0.45
	CCI	0.002	0.97	0.01	0.55
**Stretch Widthwise**					
	Age	-0.003	0.66	0.004	0.22
	Tobacco	-0.06	0.73	-0.04	0.60
	CCI	0.04	0.45	-0.02	0.38
**Epithelial Thickness**					
	Age	-0.63	0.69	-8.17	0.003*
	Tobacco	57.93	0.10	-23.62	0.71
	CCI	9.08	0.45	27.54	0.17
**Lamina Propria Thickness**					
	Age	1.46	0.30	-1.49	0.15
	Tobacco	65.4	0.04*	14.21	0.56
	CCI	0.08	0.99	7.63	0.32
**Muscularis Mucosa Thickness**					
	Age	0.46	0.13	–	–
	Tobacco	15.16	0.02*	–	–
	CCI	-5.36	0.02*	–	–
**Submucosal Thickness**					
	Age	6.55	0.37	1.12	0.88
	Tobacco	114.02	0.47	-169.00	0.334
	CCI	-88.80	0.11	-71.56	0.19

* Signifies statistical significance

## DISCUSSION

To our knowledge, this is the first report in the literature to investigate and compare the histological and stretch characteristics of buccal and colorectal grafts. We found that each graft type had differing characteristics that could either be potentially beneficial or detrimental to graft success. We believe that our findings are important for clinical practice as they demonstrate that colorectal mucosa may not be a perfect substitute for buccal mucosa, and that this may require consideration during surgical decision making and planning.

We found that BMGs and CMGs had similar overall thicknesses but differed significantly in the dimensions of their individual cell layers. BMGs had a significantly thicker epithelium than CMGs but a significantly thinner LP. This is similar to what has been previously reported in comparisons of oral mucosa to bladder mucosa and penile skin (20). It is also what has driven urologists to favor oral mucosal grafts over these historic alternatives, as the dimensions of these layers are likely relevant to graft take.

Buccal mucosa is a successful substitution tissue in urethral surgery because of its inherently thick elastin-rich epithelium, which makes it tough yet easy to handle, and its thin, highly vascularized LP, or subepithelial connective tissue layer, which is believed to facilitate early inosculation and imbibition (5, 21). Its high resistance and resilience to recurrent compression, stretching and shearing forces are partially explained by the lamina propria-oral epithelial interface, which is made up of connective tissue projections that increase surface area and provide resistance to overlying forces (20).

Prior literature has suggested that LP width is particularly important for graft take, as it enhances basal epithelial cell viability and facilitates neovascularization of the graft (4). The LP provides vascular support and nutrition to the overlaying cellular epithelium. It also contains immune cells from the adaptive and innate immune system (22). While grafts with LPs that are too thin may be at risk of necrosis or atrophy at the recipient site (23), ones that are too thick may have compromised neovascularization. We speculate that like bladder and penile skin grafts, the relatively thick LP of the colorectal mucosa may be disadvantageous for graft take. However, further research is needed.

Extrapolating from studies in skin grafting, we suspect that the thickness of the elastin-rich epithelial layer in mucosal grafts could impact graft contracture. Graft contracture occurs in two stages: primary and secondary contracture. Primary contracture, which refers to the immediate contraction that occurs directly after graft harvest, is due to passive recoil of elastin fibers and is directly dependent on the thickness of the elastin-rich dermis. Whereas secondary contracture, which refers to graft contraction on the wound bed, is caused by myofibroblasts deposition and is inversely related to dermal thickness (24). While additional work is needed, we suspect that, like the thicker elastin-rich dermis in full-thickness skin grafts, the thicker elastin-rich epithelium in BMGs may increase the risk of primary contracture but may mitigate the risk of secondary contracture by providing resistance to the pull of myofibroblast deposition during healing (24, 25).

Our finding that BMGs were significantly less elastic than CMGs on the back-table silicon block seems to support our hypothesis about primary graft contracture. We found that, on average, CMGs could be stretched to more than double their initial excision size on the back-table. On average a 5×2cm CMG graft, could be stretched to cover a 10.9×3.4cm defect, while a BMG could only cover a 6.1×2.2cm defect. Though little is known about the clinical importance of ex-vivo graft stretch, the significant gains in length and width in CMGs, could mean that a relatively smaller CMG could be used to cover a much larger defect than a BMG. While this characteristic would be particularly important in patients with long urethral strictures or limited oral mucosal availability, it remains unclear if CMG elasticity is durable in-vivo or if it becomes compromised by secondary graft contracture.

We also demonstrated that CMGs have an entirely extra cell layer, the muscularis mucosa (MM), compared to BMGs. This layer is relatively thin, approximating one-tenth the thickness of the epithelial and LP layers and is located deep to the LP but above the submucosa. The inner MM layer is made of a thin layer of smooth muscle. It supports and enables the mucosa to move and fold. Below it, is the submucosa – which is a thick connective tissue layer containing vasculature, lymphatics, and nerves (26). The clinical impact of this additional layer on graft ‘take’ and durability remains to be determined, however, its presence between the rich vascular LP layer and submucosal connective tissue layer, could represent a disadvantage to imbibition - which relies on passive exchange of nutrients into the LP. Therefore, more work is needed to determine the impact of this layer on graft outcomes.

While age, tobacco use, and CCI did not seem to have a correlation with graft stretch for each graft type, these covariates did appear to affect cell layer thicknesses. Age was inversely related to the epithelial thickness of buccal grafts. This was consistent with findings that we previously published in a smaller cohort (9). Increasing CCI was associated with decreased MM layer thickness in CMGs, suggesting that cellular health of this layer could be impacted by a patient's medical milieu. Tobacco use, including former and active smokers, was associated with increasing LP and MM thickness in CMGs. CMGs from smokers had significantly thicker LP layers than CMG from non-smokers, which is similar to what has previously been demonstrated in studies investigating histologic characteristics of uvular mucosa in smokers with obstructive sleep apnea (27) and supported by mouse studies demonstrating an association between smoking and an accumulation of inflammatory cells in the LPs of the small and large intestines (28, 29). We did not find a similar relationship between tobacco use and histologic changes in BMGs. This is supported by recent literature by Policastro et al., demonstrating no clear or clinically significant histologic or immunohistochemical differences in buccal grafts harvested from smokers compared to non-smokers (30). However, both studies had relatively small sample sizes and therefore may have been underpowered to detect an association. Further work is needed on this topic.

This study has several limitations. First, it is a single-center, single-surgeon, single-pathologist study. Second, not all BMGs resulted in pathology review. Third, our buccal patients tended to be younger and healthier, and our colorectal patients tended to be smokers, which could have impacted the qualities of our grafts, however we did control for these factors in our models to mitigate this risk. In addition, our cohorts were small, and the CMGs were collected from several different anatomic locations along the gastrointestinal tract. And last, graft stretch was assessed manually without accounting for applied force. In addition, this was not an outcomes study – therefore we do not have clinical outcomes data to correlate with our histologic findings.

## CONCLUSIONS

Our study is the first to compare the histologic properties and stretch characteristics of buccal and colorectal mucosal grafts. It also raises the question of whether certain demographic and clinical factors should influence surgeon-decision making in selecting graft type. Though more work is needed, our findings suggest that buccal grafts may continue to be a more suitable graft for urethroplasty due to their relatively thicker epithelium and thinner LP. That said, the significant elasticity of colorectal grafts may make colorectal grafts a more suitable options in patients with longer, more complex urethral strictures, or in patients who have limited oral graft availability or oral pathology. However, the durability of this elasticity during healing remains unknown. In-vivo studies, either in animal models or humans, are needed to determine if graft selection and histologic properties affect graft take, urethroplasty outcomes and the risk of stricture recurrence.
